# Individual-level income and out-of-hospital cardiac arrest survival in men and women

**DOI:** 10.1136/openhrt-2022-002044

**Published:** 2022-08-19

**Authors:** Laura H van Dongen, Robin L A Smits, Irene G M van Valkengoed, Petra Elders, Hanno L Tan, Marieke T Blom

**Affiliations:** 1 Department of Experimental Cardiology, Amsterdam UMC Locatie AMC, Amsterdam, The Netherlands; 2 Heart Failure & Arrhythmias, Amsterdam Cardiovascular Sciences, Amsterdam, The Netherlands; 3 Department of Public Health, Amsterdam UMC Locatie AMC, Amsterdam, The Netherlands; 4 Health Behaviours & Chronic Diseases, Amsterdam Public Health Research Institute, Amsterdam, The Netherlands; 5 General Practice, Amsterdam UMC Locatie VUmc, Amsterdam, The Netherlands; 6 Netherlands Heart Institute, Utrecht, The Netherlands

**Keywords:** heart arrest, epidemiology, outcome assessment, health care

## Abstract

**Objective:**

Area-level socioeconomic factors are known to associate with chances to survive out-of-hospital cardiac arrest (OHCA survival). However, the relationship between individual-level socioeconomic factors and OHCA survival in men and women is less established. This study investigated the association between individual-level income and OHCA survival in men and women, as well as its contribution to outcome variability and mediation by resuscitation characteristics.

**Methods:**

A cross-sectional cohort study using data from a Dutch community-based OHCA registry was performed. We included 5395 patients aged≥25 years with OHCA from a presumed cardiac cause. Household income, derived from Statistics Netherlands, was stratified into quartiles. The association between survival to hospital discharge and household income was analysed using multivariable logistic regression adjusting for age, sex and resuscitation characteristics.

**Results:**

Overall women had lower household income than men (median €18 567 vs €21 015), and less favourable resuscitation characteristics. Increasing household income was associated with increased OHCA survival in both men and women in a linear manner (Q4 vs Q1: OR 1.63 95% CI (1.24 to 2.16) in men, and 2.54 (1.43 to 4.48) in women). Only initial rhythm significantly changed the ORs for OHCA survival with>10% in both men and women. Household income explained 3.8% in men and 4.3% in women of the observed variance in OHCA survival.

**Conclusion:**

Both in men and women, higher individual-level household income was associated with a 1.2-fold to 2.5-fold increased OHCA survival to hospital discharge, but explained only little of outcome variability. A shockable initial rhythm was the most important resuscitation parameter mediating this association. Our results do not support the need for immediate targeted interventions on actionable prehospital resuscitation care characteristics.

**WHAT IS ALREADY KNOWN ON THIS TOPIC**

- Area-level socioeconomic factors are known to associate with out-of-hospital cardiac arrest (OHCA) survival. However, the relationship between individual-level socioeconomic factors and OHCA survival in men and women is less established, especially on the relative importance of socioeconomic status (SES) in outcome variability and mediation of resuscitation characteristics.

**WHAT THIS STUDY ADDS**

- Both in men and women, higher individual-level income was associated with increased odds to survive to hospital discharge after OHCA. Individual-level income only explained a small part of outcome variability in OHCA survival in both men and women. Initial rhythm was the most important resuscitation parameter mediating the association between income and OHCA survival in both men and women.

**HOW THIS STUDY MIGHT AFFECT RESEARCH, PRACTICE OR POLICY**

- This study does not support the need for immediate targeted interventions on actionable prehospital resuscitation care characteristics in relation to SES in both men and women.

## Introduction

Out-of-hospital cardiac arrest (OHCA) is a major public health problem, affecting 275 000 individuals in Europe each year.^1^ Survival chances after OHCA (OHCA survival) are generally low,^2^ and depend on the response system in place, resuscitation characteristics and patient characteristics.^3^ A recent systematic review indicated that several aspects of socioeconomic status (SES) impact survival chances after OHCA.^4^ Previous research has mostly focused on area-level measurements. However, recent studies have been able to use individual-level data, which better reflect the personal circumstances of an individual, and has a higher predictive value for health outcomes.^5^ Two recent studies, from Denmark and Sweden, showed that both lower income and education were associated with overall lower OHCA survival.^6 7^

To develop targeted interventions, insight into factors that drive subgroup differences in OHCA survival is imperative. Previous research has suggested differences in OHCA incidence and OHCA survival between men and women,^8–10^ and several studies suggested that women have a greater SES disparity in CVD mortality, morbidity and risk factors.^11 12^ Two previous studies suggested SES differences in OHCA survival within and between subgroups (eg, by sex) might be explained by a difference in patient and resuscitation characteristics,^8^ ^13^ as women were generally older, less likely to receive bystander cardiopulmonary resuscitation (CPR) or to have a shockable initial rhythm.^8 14^

This study aimed to investigate the association between individual-level income (personal and household) and OHCA survival to hospital discharge in men and women in the Netherlands. Moreover, the relative contribution of income to the explained variance of OHCA survival was studied to explore the importance of individual-level SES in outcome variability. Additionally, potential mediation of this association in men and women by resuscitation characteristics was investigated, as well as effect modification by age, as income typically follows a curvilinear trajectory with age.^15^

## Methods

### Setting

The Amsterdam Resuscitation Study (ARREST) is an ongoing, prospective registry of OHCA in the North Holland province of the Netherlands. The study region covers 2404 km^2^, comprises both urban and rural areas and has a population of 2.4 million people. The ARREST study group cooperates with all emergency medical services (EMS) in this study region, and registers all EMS-attended OHCAs from medical causes in which a resuscitation is attempted, according to Utstein recommendations.^16^ Details of the design of the data collection in the ARREST study are described elsewhere.^17^ The present study is a cross-sectional cohort study covering the study period 1 January 2009 to 31 December 2015. We combined data from the ARREST study with income data from Statistics Netherlands. Patients were included when they were 25 years or older, had an OHCA with a presumed cardiac cause and were residents of the Netherlands. The lower age bound was chosen under the assumption that individuals under the age of 25 were more likely to still have been in postsecondary education and not financially independent. Cases with a clear non-cardiac cause according to the Utstein recommendations,^16^ a ‘do not resuscitate’ certificate, missing SES data, missing outcome data or those who were EMS witnessed were excluded from analysis.

Written informed consent was obtained from all participants who survived the OHCA.^18^

### Patient involvement

Patients were not involved in the design and conduct of this research. Results were disseminated to patients and public through a webinar.

### Exposures

Individual-level income data were retrieved from Statistics Netherlands, which has records of yearly income and its sources, originally provided by the Dutch tax authorities. The income indicators included are: (1) personal income and (2) household income of the OHCA victim. These SES indicators were determined from the year preceding the OHCA. In brief, personal income is defined by Statistics Netherlands as all sources of gross income of a person (eg, work, own company, social benefits, excluding child benefits), minus taxes and income insurance.^19^ Household income is defined as the standardised disposable income and comprises all sources of income aggregated over the household including rebates and social benefits minus taxes and insurance premiums, corrected for household size and composition (using equivalence factors based on number of adults and children according to age in a household).^20^ Personal and household income were categorised into quartiles in the total study population (for men and women combined).

### Outcomes

The primary outcome was OHCA survival, which was defined as survival to hospital discharge. Survival status was retrieved from hospital records or from the civil registry if hospital records were unavailable (n=34, 30-day survival as proxy).

### Other measurements

Sex and age were derived from EMS, hospital records and civil registry. Resuscitation characteristics were retrieved from EMS using the Utstein definitions.^21^ The present study included the variables: location of OHCA (home vs public), response time (time from EMS call to connection of automated external defibrillator (AED) or manual defibrillator, whichever was first, in minutes), presence of witness, provision of bystander CPR, AED connection (by bystander or first responder) and initial rhythm of the patient, which was categorised as (1) shockable (ventricular tachycardia/ventricular fibrillation) or (2) non-shockable (asystole or pulseless electrical activity).

### Statistical analysis

Patient characteristics, by sex and by income quartiles, were reported as mean±SD, median (IQR) or number (percentage), where appropriate. Statistical comparisons were made using independent sample t-tests, Mann-Whitney U test or Χ^2^ statistics, where appropriate. Due to missing data in covariates, multiple imputation was performed, using the Utstein variables, demographics, outcome and exposures, employing the *‘*mice*’* package in R, resulting in five imputed datasets.

The association between household income and OHCA survival in men and women was assessed using logistic regression analysis, and repeated using personal income. Model 1 included the respective income variable and age. A final model was composed, including all resuscitation characteristics that significantly contributed to SES differences in outcome (AED connection was not included as this was highly correlated with response time). Additionally, we estimated the relative contributions of household income and personal income to OHCA survival by calculating the increase in explained variance using the Nagelkerke test (pseudo R^2^), in comparison to models without income.

To investigate potential mediation by resuscitation characteristics, models were made including income, age and one of the resuscitation characteristics each (bystander CPR, witnessed status, initial rhythm, AED use, OHCA location and response time). The change in OR (in %) across the three comparisons (Q1 vs Q2/Q3/Q4) was calculated for the addition of each resuscitation characteristic compared with model 1. Moreover, since a shockable initial rhythm is a major predictor of survival, and previous research suggested lower survival rates in women were attributed to a lower frequency of a shockable initial rhythm,^8^ a post-hoc sensitivity analysis was performed in men and women with a shockable initial rhythm (association between household income and OHCA survival).

Finally, to test whether age is an effect modifier in the association between income and OHCA survival, an interaction term (income*age both continuous) was included in the model for both men and women. Subsequently, analyses were stratified by age (younger than 65 and 65 or older). The cut-off at 65 years was chosen according to the National Old Age Pensions Act pension age.

All statistical tests were two tailed, and a p value of<0.05 was considered to be statistically significant. Statistics were performed in R V.4.0.3^22^ and SPSS V.25.0.

## Results

**Figure 1:**
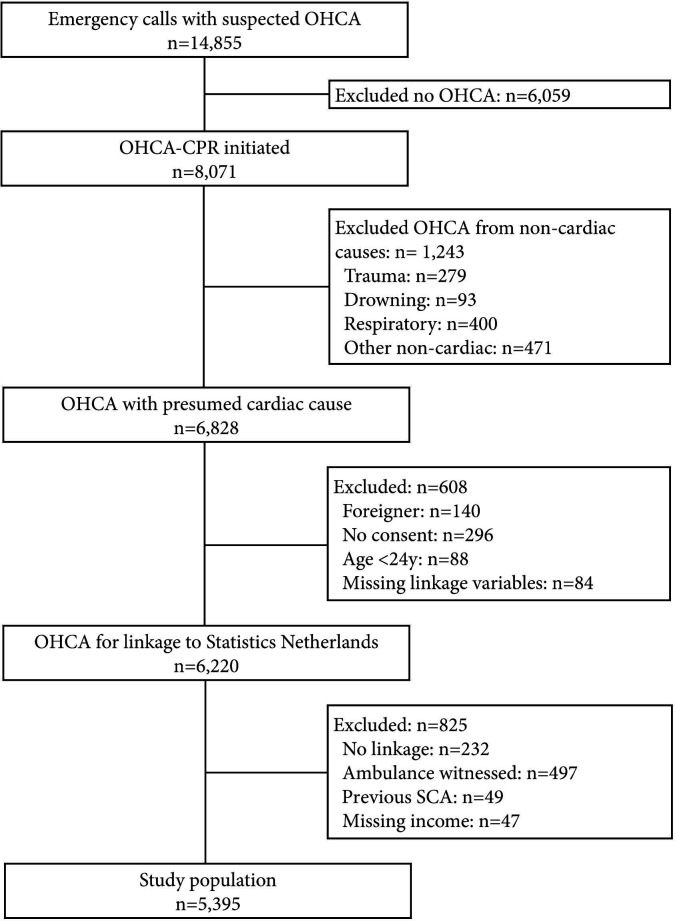
Flow chart of patient inclusion. CPR, cardiopulmonary resuscitation; OHCA, out-of-hospital cardiac arrest; SCA, sudden cardiac arrest.

During our study period, 5395 patients were eligible for inclusion (figure 1). In this study population, 71% was male and 29% was female. Overall, women were older, and had lower personal and household income as compared with men (table 1). In men, personal and household income have a linear relationship (online supplemental efigure 1). In contrast, personal income was less well correlated with household income in women, and women with no personal income appeared to have higher household income compared with women in Q2. Moreover, OHCAs in women were less frequently witnessed by a bystander, less frequently received bystander CPR, less frequently had an AED connected, were less frequently in a public location, less often had a shockable initial rhythm and had longer times to defibrillator connection as compared with men (table 1). Baseline characteristics before imputation yielded similar results (online supplemental etable 1).

10.1136/openhrt-2022-002044.supp1 Supplementary data

**Table 1:** Patient characteristics by sex

	Men, n=3829	Women, n=1566	P value
Age, mean±SD	67.23±12.89	70.37±14.09	<0.001
Personal income in €, median (IQR)*	25 166 (15 854–38 613)	13 781 (9545–19 689)	<0.001
Q1	608 (15.9)	748 (47.8)	
Q2	876 (22.9)	465 (29.7)	
Q3	1121 (29.3)	228 (14.6)	
Q4	1224 (32.0)	125 (8.0)	
Household income in €, median (IQR)*	21 015 (16 553–28 315)	18 567 (15 353–24 562)	<0.001
Q1	859 (22.4)	489 (31.2)	
Q2	921 (24.1)	428 (27.3)	
Q3	985 (25.7)	364 (23.2)	
Q4	1064 (27.8)	285 (18.2)	
Resuscitation characteristics			
Witnessed arrest	2780 (72.6)	1070 (68.4)	0.002
Bystander CPR	3039 (79.4)	1193 (76.2)	0.011
AED used	2103 (54.9)	800 (51.1)	0.010
Public location	1178 (30.8)	230 (14.7)	<0.001
Shockable initial rhythm	2014 (52.6)	478 (30.5)	<0.001
Response time, median (IQR)	8.4 (6.5, 10.8)	8.8 (7.0, 11.3)	<0.001
Number (proportion) of survivors	846 (22.1)	210 (13.4)	<0.001

Results are after imputation and presented as n(%), unless indicated otherwise.

*Quartiles were made based on the total population—men and women combined.

AED, automated external defibrillator; CPR, cardiopulmonary resuscitation.

Resuscitation characteristics varied across household income quartiles (table 2). For instance, with increasing quartiles of household income, patients were more likely to have a bystander witness the arrest, bystander CPR, public location of the OHCA, a shockable initial rhythm and shorter response time, but AED connection rate was comparable across quartiles. Results were similar for personal income (online supplemental etable 2).

**Table 2:** Patient characteristics by household income quartiles

	Household income Q1 (€0–16.097) n=1348	Household income Q2 (€16.100–20.189) n=1349	Household income Q3 (€20.190–27.290) n=1349	Household income Q4 (€27.301–255.244) n=1349	P value
Age in years, mean±SD	68.6±14.4	71.4±12.5	67.8±13.2	64.1±12.2	<0.001
Female sex	489 (36.3)	428 (31.7)	364 (27.0)	285 (21.1)	<0.001
Resuscitation characteristics					
Witnessed arrest	908 (67.4)	969 (71.8)	999 (74.1)	974.4 (72.2)	0.001
Bystander CPR	1018.6 (75.6)	1052.2 (78.0)	1050.2 (77.9)	1111.2 (82.4)	<0.001
AED connected	708.4 (52.6)	736 (54.6)	697 (51.7)	762 (56.5)	0.057
Public location	277.6 (20.6)	306 (22.7)	363 (26.9)	461 (34.2)	<0.001
Shockable initial rhythm	469.8 (34.9)	566.2 (42.0)	669.6 (49.6)	786.2 (58.3)	<0.001
Response time, median (IQR)	8.7 (6.8–11.2)	8.7 (6.7–10.8)	8.6 (6.7–10.9)	8.2 (6.4–10.6)	<0.001
Number (proportions) of survivors, total	152 (11.3)	215 (15.9)	303 (22.5)	386 (28.6)	<0.001
Men	116 (13.5)	164 (17.8)	242 (24.6)	324 (30.5)	<0.001
Women	36 (7.4)	51 (11.9)	61 (16.8)	62 (21.8)	<0.001

Results are after imputation and presented as n(%), unless indicated otherwise.

AED, automated external defibrillator; CPR, cardiopulmonary resuscitation.

**Figure 2:**
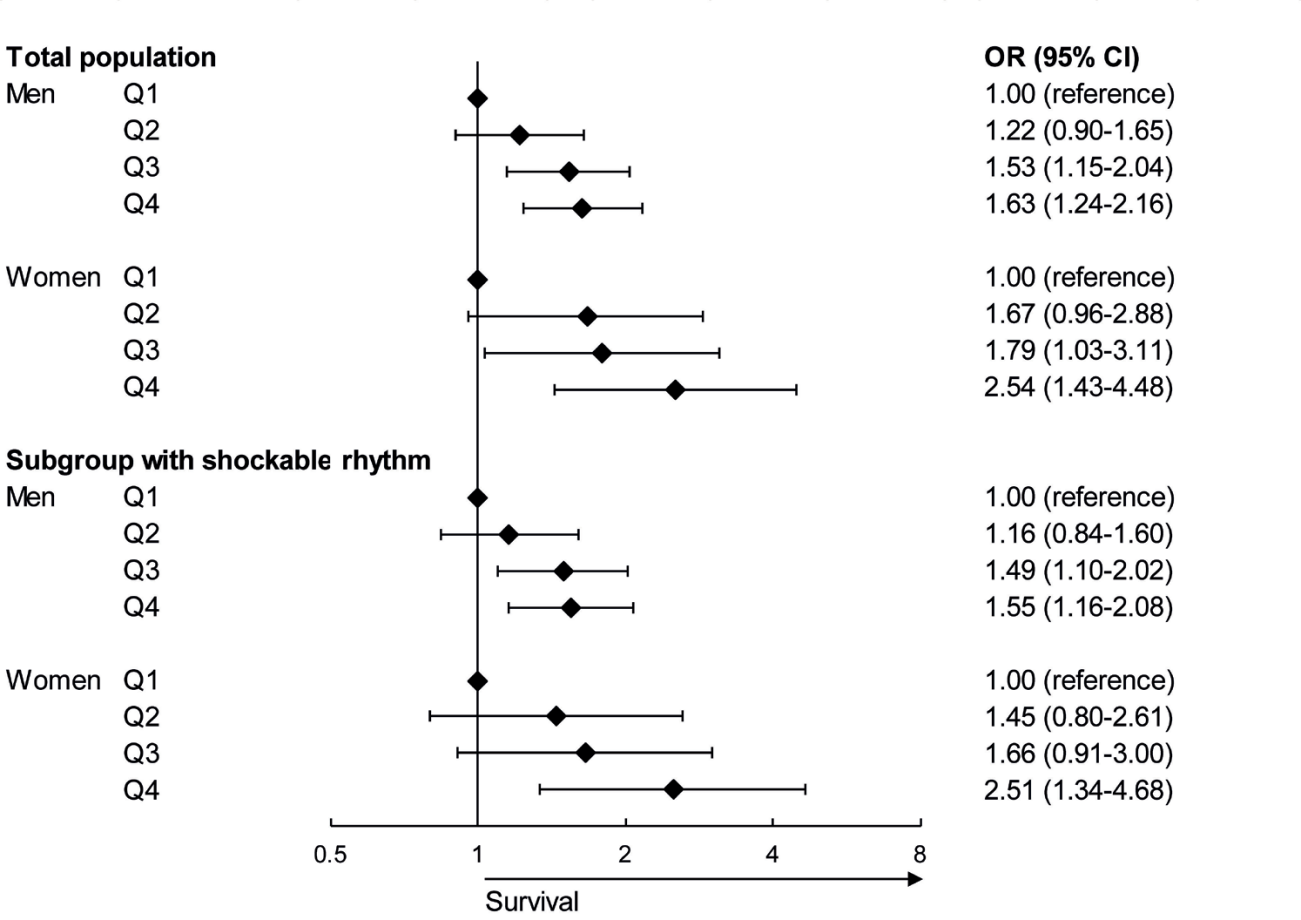
Forest plot of the association between household income and out-of-hospital cardiac arrest (OHCA) survival in the fully adjusted model in men and women in the total population and a subgroup with shockable initial rhythm. The model adjusted for age and resuscitation characteristics (witnessed by a bystander, provision of bystander cardiopulmonary resuscitation, OHCA location and time to defibrillator connection).

In both men and women, a linear correlation was observed between increasing household income quartiles and OHCA survival in our full multivariable model: compared with Q1, men and women in Q3 and Q4 were significantly more likely to survive (figure 2). However, CIs of Q2–Q4 overlapped. Trends were similar for personal income (online supplemental efigure 2). In a model only including household income, household income explained 3.8% in men and 4.3% in women of the observed variance (personal income: 4.2% in men and 3.7% in women). A model including only resuscitation characteristics explained 41.0% in men and 48.3% in women of the observed variance in the association with OHCA survival. In the full model, including household income, age and resuscitation characteristics, the explained variance was 43.1% in men and 54.0% in women (personal income: 42.8% in men and 54.1% in women). In women, the explained variance was approximately 11% higher as compared with men in the fully adjusted model.

### Mediation by resuscitation characteristics

In both men and women, the addition of initial rhythm to a model including household income and age significantly changed the ORs for OHCA survival with >10% across the three comparisons (Q1 vs Q2/Q3/Q4), but the addition of witnessed arrest, bystander CPR, OHCA location, time to defibrillator connection and AED connection did not (online supplemental etable 3, personal income: online supplemental etable 4). Subgroup analysis in men and women with a shockable initial rhythm showed small decreases in ORs in our fully adjusted model (Q1 vs Q4: from 1.63 (1.24–2.16) to 1.55 (1.16–2.08) in men, and from 2.54 (1.43–4.48) to 2.51 (1.34–4.68) in women, figure 2; similar results in personal income, online supplemental efigure 2).

### Effect modification by age

**Figure 3:**
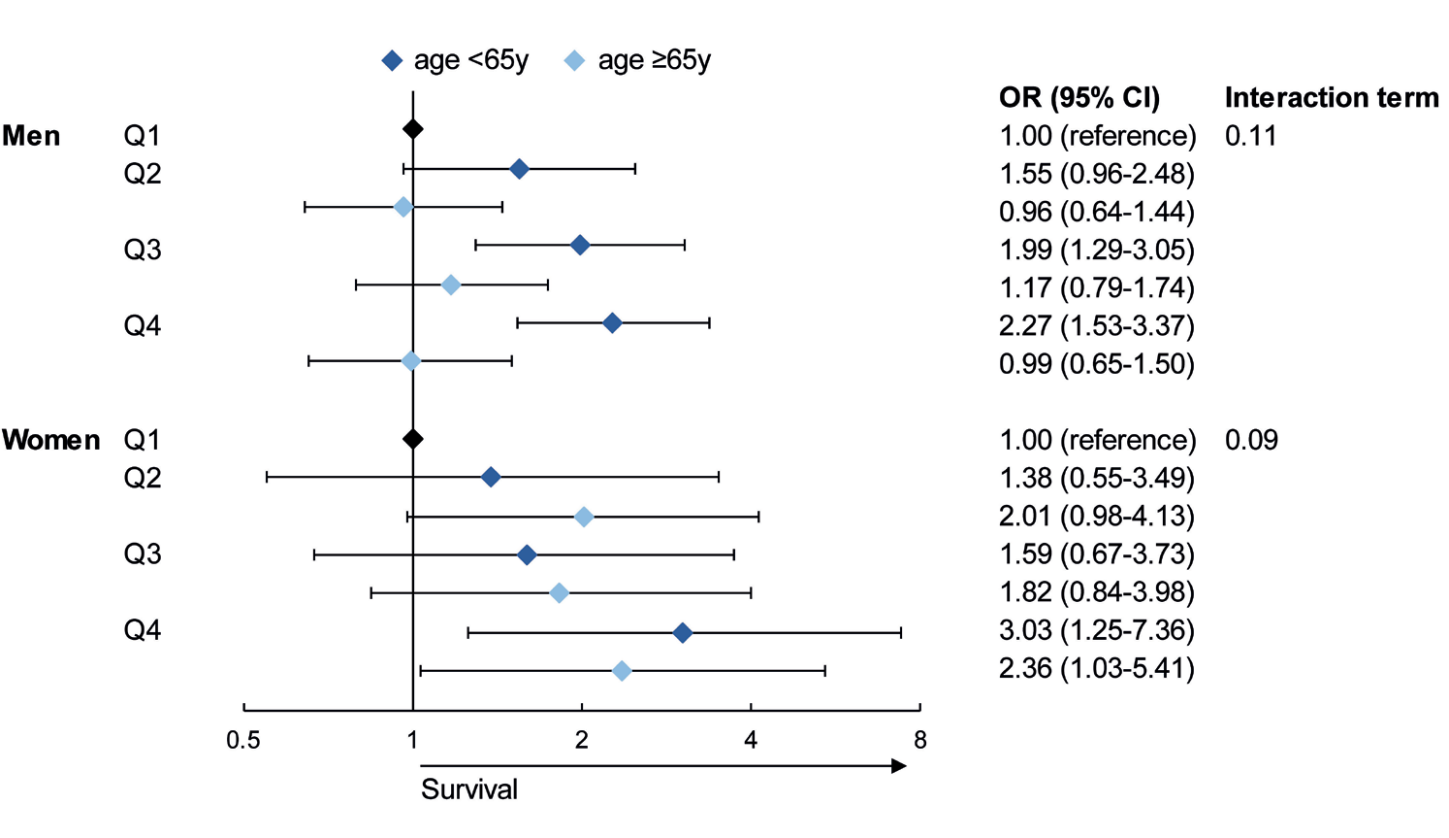
Forest plot of the association between household income and out-of-hospital cardiac arrest (OHCA) survival in the fully adjusted model in men and women stratified by age. The model adjusted for age and resuscitation characteristics (witnessed by a bystander, provision of bystander cardiopulmonary resuscitation, OHCA location and time to defibrillator connection).

Distribution of household income across age was more comparable between men and women, as compared with personal income across age (online supplemental efigures 3 and 4). In our full multivariable model, men aged<65 years showed increasing OHCA survival with increasing quartiles of household income, but this was not seen in men aged≥65 (figure 3, personal income online supplemental efigure 5). Both women aged<65 and ≥65 years showed increasing odds for survival benefits with increasing quartiles, only significant in Q4. However, the CIs were wide, and the association between income and OHCA survival was not significantly different between age groups (p interaction: 0.11 in men and 0.09 in women).

## Discussion

In our study population, a higher individual-level income in men and women was associated with a 1.2-fold to 2.5-fold increased OHCA survival. Nevertheless, income only contributed to a small increase in the explained variance in men and women. In both sexes, initial rhythm partly mediated the association between income and OHCA survival, but other resuscitation parameters did not. Findings were similar for personal income, and in a subgroup of patients with a shockable initial rhythm. In men younger than 65 years increasing income was associated with increased OHCA survival, but in men aged 65 years or older it was not, though the interaction was not statistically significant. In women, no age difference was observed.

Consistent with two previous Danish and Swedish studies,^6 7^ we observed a statistically significant association between household income and OHCA survival in men and women. This is surprising because the Netherlands is a country with relatively high and evenly distributed prosperity (Legatum Prosperity Index 2020=82 [ranked sixth], Gini index for income=0.29) and high rates of basic life support-trained individuals and AED connections. Consequently, other countries with different care systems and greater prosperity disparity might observe even larger differences.

Prior work on, mostly area-level, income disparities and OHCA survival have described the importance to improve of bystander response rates (CPR and/or AED connection) in lower income areas.^4 23^ Recent work showed that adjusting for SES differences did not mitigate outcome differences in bystander CPR,^6 24^ which is supported by results from this study. Although patients with higher income more frequently had favourable resuscitation characteristics (except AED connection rate), these resuscitation characteristics did not fully explain the association between SES and OHCA survival. In the Netherlands, AEDs are mostly connected by dispatched first responders (police or firefighters) instead of citizen responders or bystanders who happen to witness the OHCA.^25^ In a situation without these first responders with an AED, an SES gradient might be more apparent, as previous research showed that areas with low SES have lower density of citizen responders and lower connection rates of public AEDs.^26^ Initial rhythm was the principal mediator in the association between household income and OHCA survival. However, in our subgroup analysis in patients with a shockable initial rhythm, the association between income and OHCA survival remained. The fact that shockable initial rhythm was shown to be the largest mediator was not surprising, as the gradient of shockable initial rhythm over income quartiles was large and shockable initial rhythm is the major predictor for OHCA survival. This may be explained by a higher proportion of other favourable resuscitation characteristics with increased income, for example, bystander witnessed arrest, public location and a shorter response time. Although these factors played a lesser role in the association between household income and OHCA survival, they may be important for the presence of a shockable initial rhythm. Working status may be associated with the increased proportions of favourable resuscitation characteristics, as individuals who work have a higher income, and are more often in a public location and therefore more likely will have a witnessed collapse and receive bystander CPR, with subsequent higher chance of survival.

The mechanism by which socioeconomic factors may influence OHCA outcomes are yet unclear; in both men and women, resuscitation characteristics do not explain the association between income and OHCA survival. A direct causal relationship between income and OHCA survival is unlikely from a biological perspective. It is more likely that SES is a proxy, for example, accumulated risk throughout life^27^ or lifestyle behaviours.^28 29^ Two previous studies on income and OHCA survival that adjusted for several comorbidities still observed SES differences in OHCA survival,^6 7^ indicating that unmeasured factors, for example, lifestyle, might play a role. Moreover, although our analyses were adjusted for resuscitation characteristics, disparities in other cardiac arrest related factors could affect outcome as, for example, the recognition of cardiac arrest and subsequent time to interventions.^6^ This is supported by our finding that a large difference in the frequency of shockable initial rhythm is observed between income quartiles.

Overall, we found consistent associations between income and OHCA survival across age in men and women, except in older men. Survivorship bias may have attenuated results in the older men, as individuals with worse survival odds for OHCA may have died of other causes due to conditions associated with lower SES, resulting in a healthier comparator (Q1). However, in older women we did observe an association between increasing income and survival. Survivorship bias may have had a lesser effect in women as they overall have a higher life expectancy and have reported lower cardiovascular mortality rates.^30–32^ Our results are different to a previous study that investigated age differences in income and OHCA survival in Denmark.^6^ Møller *et al* found an opposite trend in the 30–65 years population as compared with ≥65 years population.

### Implications

This study does not support the need for immediate targeted interventions on actionable prehospital resuscitation care characteristics in relation to SES in the Netherlands, since actionable resuscitation characteristics did not explain the association between income and OHCA survival. Nevertheless, interventions targeted at SES-related factors, for example, lifestyle, might be useful. Though more research is required, for example, by studying the association between income and survival after OHCA in other countries, as well as by getting more insight into the living circumstances and lifestyle habits of study populations.

### Limitations

First, income in the year preceding the year of OHCA was used to determine an individual’s SES. This might not fully grasp SES over time, as income might have changed in the years before OHCA, leading to misclassification and reducing associations. Furthermore, household income was not linearly related with personal income in women. The lowest quartile of personal income contained women with relatively high household income (Q3 of Q4). Therefore, Q1 of personal income in women may contain noise. This may be explained by women more often staying at home with their partner being the primary earner.^15^ Therefore, we choose to focus on household income, as this may be a better indicator of SES when comparing the association between SES and OHCA survival between men and women. Nonetheless, using household income assumes an even distribution of income according to the needs in the household, which may not always be true.^15^ Also, the association between income and survival may be influenced by the healthy worker effect, the phenomenon that healthy individuals are more likely to be represented in the workforce, and therefore have a (higher) income, compared with sicker or less abled individuals.^33^ Nevertheless, household income is a measure that is corrected for household size and composition, which is less affected by the healthy worker effect as compared with personal income. Furthermore, patients that could not be linked to Statistics Netherlands had to be excluded. Since, only 5% could not be matched and missing case analysis did not show significant differences in characteristics with the linked cases, we do not expect that this has led to substantial selection bias. Moreover, in the dataset available for this study, we did not have comorbidity data available. Nevertheless, a Danish study from Møller *et al*,^6^ showed that the association between SES and OHCA survival persisted after adjusting for comorbidities. We expect that the same would apply for our study population, given the similarities between the study populations. Additionally, differences in hospital treatment might exist and affect survival, but these data are unavailable. Lastly, residual confounding may still exist despite our attempts to control for possible confounding factors, and the nature of this study does not allow for causal inference.

### Conclusion

Both in men and women, higher individual-level income was associated with increased odds to survive to hospital discharge after OHCA. Individual-level income only explained a small part of outcome variability in OHCA survival in both men and women. A shockable initial rhythm was the most important resuscitation parameter mediating the association between income and OHCA survival in both men and women. Therefore, this study does not support the need for immediate targeted interventions on actionable prehospital resuscitation care characteristics in relation to SES in both men and women.

## Data Availability

All data relevant to the study are included in the article or uploaded as supplemental information. The data cannot be shared publicly for privacy of individuals that participated in the study as data cannot be provided completely anonymous according to the Medical Ethics Committee and the Data Protection Officer of our institution (MEC: mecamc@amc.nl, DPO: fg@amc.nl).

## References

[1] Gräsner J-T, Lefering R, Koster RW, et al. EuReCa ONE-27 nations, one Europe, one registry: a prospective one month analysis of out-of-hospital cardiac arrest outcomes in 27 countries in Europe. Resuscitation 2016;105:188–95. 10.1016/j.resuscitation.2016.06.004 27321577

[2] Gräsner J-T, Wnent J, Herlitz J, et al. Survival after out-of-hospital cardiac arrest in Europe - results of the EuReCa TWO study. Resuscitation 2020;148:218–26. 10.1016/j.resuscitation.2019.12.042 32027980

[3] Gräsner J-T, Herlitz J, Tjelmeland IBM, et al. European resuscitation council guidelines 2021: epidemiology of cardiac arrest in Europe. Resuscitation 2021;161:61–79. 10.1016/j.resuscitation.2021.02.007 33773833

[4] van Nieuwenhuizen BP, Oving I, Kunst AE, et al. Socio-economic differences in incidence, bystander cardiopulmonary resuscitation and survival from out-of-hospital cardiac arrest: a systematic review. Resuscitation 2019;141:44–62. 10.1016/j.resuscitation.2019.05.018 31199944

[5] Wells DM, White LLY, Fahrenbruch CE, et al. Socioeconomic status and survival from ventricular fibrillation out-of-hospital cardiac arrest. Ann Epidemiol 2016;26:418–23. 10.1016/j.annepidem.2016.04.001 27174737

[6] Møller S, Wissenberg M, Starkopf L, et al. Socioeconomic disparities in prehospital factors and survival after out-of-hospital cardiac arrest. Heart 2021;107:627–34. 10.1136/heartjnl-2020-317761 33419881

[7] Jonsson M, Härkönen J, Ljungman P, et al. Inequalities in income and education are associated with survival differences after out-of-hospital cardiac arrest: nationwide observational study. Circulation 2021;144:1915–25. 10.1161/CIRCULATIONAHA.121.056012 34767462 PMC8663522

[8] Blom MT, Oving I, Berdowski J, et al. Women have lower chances than men to be resuscitated and survive out-of-hospital cardiac arrest. Eur Heart J 2019;40:3824–34. 10.1093/eurheartj/ehz297 31112998 PMC6911168

[9] Ro YS, Shin SD, Song KJ, et al. A trend in epidemiology and outcomes of out-of-hospital cardiac arrest by urbanization level: a nationwide observational study from 2006 to 2010 in South Korea. Resuscitation 2013;84:547–57. 10.1016/j.resuscitation.2012.12.020 23313428

[10] Luc G, Baert V, Escutnaire J, et al. Epidemiology of out-of-hospital cardiac arrest: a French national incidence and mid-term survival rate study. Anaesth Crit Care Pain Med 2019;38:131–5. 10.1016/j.accpm.2018.04.006 29684654

[11] Mackenbach JP, Cavelaars AE, Kunst AE, et al. Socioeconomic inequalities in cardiovascular disease mortality; an international study. Eur Heart J 2000;21:1141–51. 10.1053/euhj.1999.1990 10924297

[12] Backholer K, Peters SAE, Bots SH, et al. Sex differences in the relationship between socioeconomic status and cardiovascular disease: a systematic review and meta-analysis. J Epidemiol Community Health 2017;71:550–7. 10.1136/jech-2016-207890 27974445

[13] Vaillancourt C, Lui A, De Maio VJ, et al. Socioeconomic status influences bystander CPR and survival rates for out-of-hospital cardiac arrest victims. Resuscitation 2008;79:417–23. 10.1016/j.resuscitation.2008.07.012 18951678

[14] van Dongen LH, Oving I, Dijkema PW, et al. Sex differences in the association of comorbidity with shockable initial rhythm in out-of-hospital cardiac arrest. Resuscitation 2021;167:173–9. 10.1016/j.resuscitation.2021.08.034 34455022

[15] Galobardes B, Shaw M, Lawlor DA, et al. Indicators of socioeconomic position (part 1). J Epidemiol Community Health 2006;60:7–12. 10.1136/jech.2004.023531 PMC2465546 16361448

[16] Jacobs I, Nadkarni V, Bahr J, et al. Cardiac arrest and cardiopulmonary resuscitation outcome reports: update and simplification of the Utstein templates for resuscitation registries. A statement for healthcare professionals from a task force of the International liaison Committee on resuscitation (American heart association, European resuscitation Council, Australian resuscitation Council, New Zealand resuscitation Council, heart and stroke Foundation of Canada, InterAmerican heart Foundation, resuscitation Council of southern Africa). Resuscitation 2004;63:233–49. 10.1016/j.resuscitation.2004.09.008 15582757

[17] Blom MT, van Hoeijen DA, Bardai A, et al. Genetic, clinical and pharmacological determinants of out-of-hospital cardiac arrest: rationale and outline of the Amsterdam resuscitation studies (arrest) registry. Open Heart 2014;1:e000112. 10.1136/openhrt-2014-000112 25332818 PMC4189338

[18] Bak MAR, Blom MT, Tan HL, et al. Ethical aspects of sudden cardiac arrest research using observational data: a narrative review. Crit Care 2018;22:212. 10.1186/s13054-018-2153-3 30208954 PMC6136218

[19] Centraal Bureau voor de Statistiek. Documentatie Integraal Persoonlijk Inkomen (IPI). Den Haag: Microdata Services, 2018.

[20] Centraal Bureau voor de Statistiek. Documentatie Inkomen van huishoudens (INHATAB). Den Haag: Microdata Services, 2021.

[21] Perkins GD, Jacobs IG, Nadkarni VM, et al. Cardiac arrest and cardiopulmonary resuscitation outcome reports: update of the Utstein resuscitation registry templates for out-of-hospital cardiac arrest: a statement for healthcare professionals from a task force of the International liaison Committee on resuscitation (American heart association, European resuscitation Council, Australian and New Zealand Council on resuscitation, heart and stroke Foundation of Canada, InterAmerican heart Foundation, resuscitation Council of southern Africa, resuscitation Council of Asia); and the American heart association emergency cardiovascular care Committee and the Council on cardiopulmonary, critical care, perioperative and resuscitation. Resuscitation 2015;96:328–40. 10.1016/j.resuscitation.2014.11.002 25438254

[22] Team; RC. R: A language and environment for statistical computing (version 3.5. 2, R foundation for statistical computing, Vienna, Austria, 2018) 2019.

[23] Huebinger R, Vithalani V, Osborn L, et al. Community disparities in out of hospital cardiac arrest care and outcomes in Texas. Resuscitation 2021;163:101–7. 10.1016/j.resuscitation.2021.03.021 33798624

[24] Chan PS, McNally B, Vellano K, et al. Association of neighborhood race and income with survival after out-of-hospital cardiac arrest. J Am Heart Assoc 2020;9:e014178. 10.1161/JAHA.119.014178 32067590 PMC7070200

[25] Stieglis R, Zijlstra JA, Riedijk F, et al. AED and text message responders density in residential areas for rapid response in out-of-hospital cardiac arrest. Resuscitation 2020;150:170–7. 10.1016/j.resuscitation.2020.01.031 32045663

[26] Andersen LW, Holmberg MJ, Granfeldt A, et al. Neighborhood characteristics, bystander automated external defibrillator use, and patient outcomes in public out-of-hospital cardiac arrest. Resuscitation 2018;126:72–9. 10.1016/j.resuscitation.2018.02.021 29477731

[27] Huijts T, Eikemo TA. Causality, social selectivity or artefacts? Why socioeconomic inequalities in health are not smallest in the Nordic countries. Eur J Public Health 2009;19:452–3. 10.1093/eurpub/ckp103 19587229

[28] Jakobsen L, Niemann T, Thorsgaard N, et al. Dimensions of socioeconomic status and clinical outcome after primary percutaneous coronary intervention. Circ Cardiovasc Interv 2012;5:641–8. 10.1161/CIRCINTERVENTIONS.112.968271 23031837

[29] Loh VHY, Rachele JN, Brown WJ, et al. Neighborhood disadvantage, individual-level socioeconomic position and physical function: a cross-sectional multilevel analysis. Prev Med 2016;89:112–20. 10.1016/j.ypmed.2016.05.007 27196142

[30] Bots SH, Peters SAE, Woodward M. Sex differences in coronary heart disease and stroke mortality: a global assessment of the effect of ageing between 1980 and 2010. BMJ Glob Health 2017;2:e000298. 10.1136/bmjgh-2017-000298 PMC5435266 28589033

[31] Mikkola TS, Gissler M, Merikukka M, et al. Sex differences in age-related cardiovascular mortality. PLoS One 2013;8:e63347. 10.1371/journal.pone.0063347 23700418 PMC3658978

[32] Rogers RG, Everett BG, Onge JMS, et al. Social, behavioral, and biological factors, and sex differences in mortality. Demography 2010;47:555–78. 10.1353/dem.0.0119 20879677 PMC3000060

[33] Baillargeon J. Characteristics of the healthy worker effect. Occup Med 2001;16:359–66. 11319057

