# Correction: *Individual-level income and out-of-hospital cardiac arrest survival in men and women*

**DOI:** 10.1136/openhrt-2022-002044corr1

**Published:** 2022-11-15

van Dongen LH, Smits RLA, van Valkengoed IGM, *et al*. Individual-level income and out-of-hospital cardiac arrest survival in men and women. *Open Heart* 2022;9:e002044. doi: 10.1136/openhrt-2022-002044

Author names Laura H van Dongen and Hanno L Tan have been updated to include their middle initials.

