# Supplementary material for: Individual-level income and out-of-hospital cardiac arrest survival in men and women
Source: Open Heart. 2022 Aug 19;9(2):e002044. doi: 10.1136/openhrt-2022-002044 (PMC9396148; doi:10.1136/openhrt-2022-002044)

eTable 1. Baseline characteristics in men and women before multiple imputation.

	Men n=3,829	Women n=1,566	P-value	Missing values
Age, mean±SD	67.23 ± 12.89	70.37 ± 14.09	<0.001	0%
Median personal income, € (IQR)	25,166 (15,854-38,613)	13,781 (9,545- 19,689)	<0.001	0%
Q1	608 (15.9)	748 (47.8)		
Q2	876 (22.9)	465 (29.7)		
Q3	1121 (29.3)	228 (14.6)		
Q4	1224 (32.0)	125 (8.0)		
Median disposable household income, € (IQR)	21,015 (16,553- 28,315)	18,567 (15,353- 24,562)	<0.001	0%
Q1	859 (22.4)	489 (31.2)		
Q2	921 (24.1)	428 (27.3)		
Q3	985 (25.7)	364 (23.2)		
Q4	1,064 (27.8)	285 (18.2)		
Resuscitation characteristics				
Witnessed arrest	2,750 (71.8)	1,059 (67.6)	0.007	1.1%
Bystander CPR	3,014 (78.7)	1,178 (75.2)	0.006	1.5%
AED used	2,103 (54.9)	800 (51.1)	0.031	0.04%
Public location	1,177 (30.7)	230 (14.7)	<0.001	0.04%
Shockable initial rhythm	1,977 (51.6)	467 (29.8)	<0.001	2.4%
Response time, median (IQR)	8.4 (6.5, 10.7)	8.8 (6.9, 11.2)	<0.001	8.7%

Results are presented as n(%), unless indicated otherwise.

Abbreviations: AED=automated external defibrillator, CPR=cardiopulmonary resuscitation, EMS=emergency medical service.

eTable 2. Baseline characteristics by personal income quartiles.

	Personal income Q1 (€ 0-13.543) n=1356	Personal income Q2 (€ 13.544-20.746) n=1341	Personal income Q3 (€ 20.749-33.995) n=1349	Personal income Q4 (€ 33.996-792.309) n=1349	P
Age in years, mean±.D	70.8 ± 12.7	71.1 ± 13.6	68.6 ± 12.8	62.0 ± 12.1	<0.001
Female sex	748 (55.2)	465 (34.7)	228 (16.9)	125 (9.3)	<0.001
Household income					<0.001
Q1 (€ 0-16.097)	673 (49.6)	546 (40.7)	114 (8.5)	15 (1.1)	
Q2 (€ 16.100-20.189)	338 (24.9)	546 (40.7)	388 (28.8)	77 (5.7)	
Q3 (€ 20.190-27.290)	226 (16.7)	163 (12.2)	612 (45.4)	348 (25.8)	
Q4 (€ 27.301-255.244)	119 (8.8)	86 (6.4)	235 (17.4)	909 (67.4)	
Resuscitation characteristics					
Witnessed arrest	963.6 (71.1)	922 (68.8)	977.4 (72.5)	987.4 (73.2)	0.058
Bystander CPR	1008.4 (74.4)	1024.8 (76.4)	1071.2 (79.4)	1127.8 (83.6)	<0.001
AED connected	693 (51.1)	709.4 (52.9)	745 (55.2)	756 (56)	0.42
Public location	212.6 (15.7)	284 (21.2)	379 (28.1)	532 (39.4)	<0.001
Shockable initial rhythm	465.8 (34.4)	495 (36.9)	686.4 (50.9)	844.2 (62.6)	<0.001
Response time, median (IQR)	8.9 (7.1-11.4)	8.7 (6.7-11.0)	8.4 (6.5-10.8)	8.1 (6.3-10.6)	<0.001
Survival rate, total	180 (13.3)	168 (12.5)	296 (21.9)	412 (30.5)	<0.001
Men	89 (14.6)	127 (14.5)	250 (22.3)	380 (31.0)	<0.001
Women	91 (12.2)	41 (8.8)	46 (20.2)	32 (25.6)	<0.001

Results are presented as n(%), unless indicated otherwise.

Abbreviations: AED=automated external defibrillator, CPR=cardiopulmonary resuscitation, EMS=emergency medical service.

eTable 3. Mediation of resuscitation factors in the association between household income and OHCA survival chances.

		Household income quartiles					
		Q2		Q3		Q4	
		OR	% change	OR	% change	OR	% change
Men	Model 1	1.63		2.24		2.74	
	Model 1 + initial rhythm	1.34*	-17.8%	1.66*	-25.9%	1.76*	-35.8%
	Model 1 + bystander witnessed	1.54	-5.5%	2.14	-4.5%	2.69	-1.8%
	Model 1 + bystander CPR	1.59	-2.5%	2.18	-2.7%	2.59	-5.5%
	Model 1 + OHCA location	1.53	-6.1%	2.07	-7.6%	2.41*	-12.0%
	Model 1 + response time	1.58	-3.1%	2.22	-0.9%	2.62	-4.4%
	Model 1 + AED connected	1.61	-1.2%	2.26	0.9%	2.71	-1.1%
Women	Model 1	1.92		2.23		2.88	
	Model 1 + initial rhythm	1.57*	-18.2%	1.74*	-22.0%	2.52*	-12.5%
	Model 1 + bystander witnessed	1.87	-2.6%	2.12	-4.9%	2.75	-4.5%
	Model 1 + bystander CPR	1.88	-2.1%	2.29	2.7%	2.92	1.4%
	Model 1 + OHCA location	1.99	3.6%	2.37	6.3%	2.71	-5.9%
	Model 1 + response time	1.96	2.1%	2.26	1.3%	2.90	0.7%
	Model 1 + AED connected	1.95	1.6%	2.30	3.1%	2.92	1.4%

* indicates a change of >10%

Model 1 included household income (quartiles) and age (continuous).

AED=automated external defibrillator, CPR=cardiopulmonary resuscitation, OHCA=out-of-hospital cardiac arrest, OR=odds ratio.

eTable 4. Mediation of resuscitation factors in the association between personal income and OHCA survival.

		Personal income quartiles					
		Q2		Q3		Q4	
		OR	% change	OR	% change	OR	% change
Men	Model 1	1		1.61		2.14	
	Model 1 + initial rhythm	0.98	-2.0%	1.2	-25.5%	1.37	-36.0%
	Model 1 + bystander witnessed	1.03	3.0%	1.6	-0.6%	2.14	0.0%
	Model 1 + bystander CPR	0.98	-2.0%	1.56	-3.1%	2.04	-4.7%
	Model 1 + OHCA location	0.95	-5.0%	1.46	-9.3%	1.75	-18.2%
	Model 1 + response time	0.97	-3.0%	1.54	-4.4%	2.02	-5.6%
	Model 1 + AED connected	1.01	1.0%	1.61	0.00%	2.16	0.9%
Women	Model 1	0.66		1.64		1.61	
	Model 1 + initial rhythm	0.66	0.00%	1.82	11.0%	1.69	5.0%
	Model 1 + bystander witnessed	0.73	10.6%	1.81	10.4%	1.71	6.2%
	Model 1 + bystander CPR	0.65	-1.5%	1.59	-3.1%	1.54	-4.4%
	Model 1 + OHCA location	0.59	-10.6%	1.39	-15.2%	1.33	-17.4%
	Model 1 + response time	0.65	-1.5%	1.63	-0.6%	1.58	-1.9%
	Model 1 + AED connected	0.64	-3.0%	1.59	-3.1%	1.58	-1.9%

Model 1 included personal income (quartiles) and age (continuous). The green marked cells show a 10% change of the OR as compared to the model only including income and age.

AED=automated external defibrillator, CPR=cardiopulmonary resuscitation, OHCA=out-of-hospital cardiac arrest, OR=odds ratio.

In both men and women, OHCA location significantly changed the odds ratio for OHCA survival with >10% across the three comparisons (Q1 vs Q2/Q3/Q4), but initial rhythm only changed the odds ratio with >10% in men.

eFigure 1. Distribution of household income over personal income in men and women.

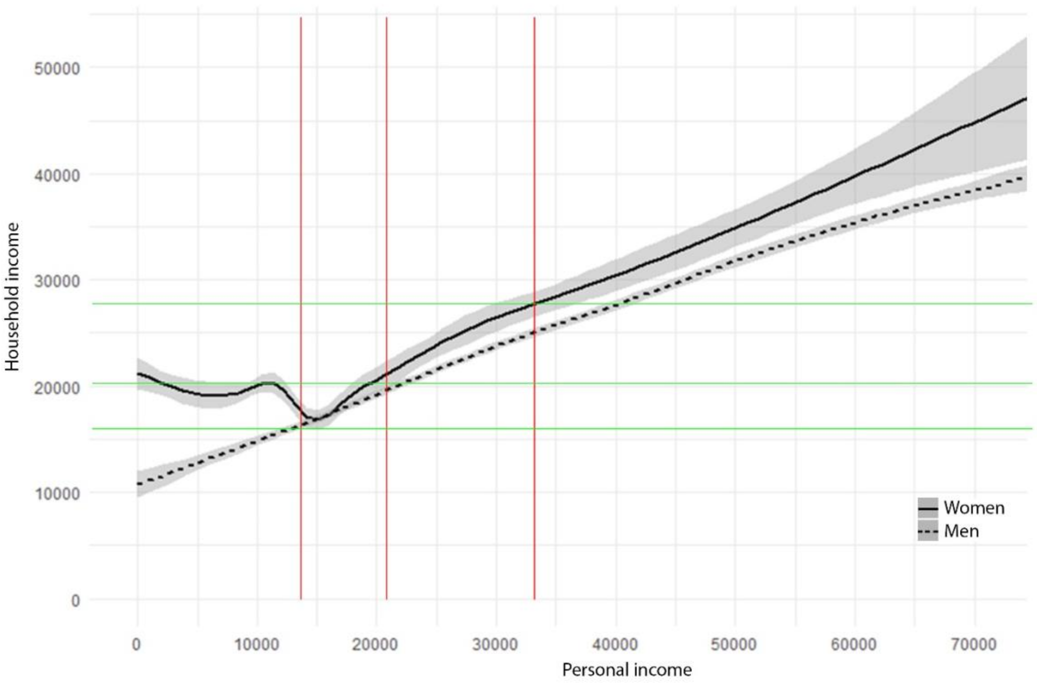

The red lines represent the borders of the quartiles of personal income.

The green lines represent the borders of the quartiles of household income.

eFigure 2. Forest plot of the association between personal income and OHCA survival in the fully adjusted model in men and women in the total population and a subgroup with shockable initial rhythm.

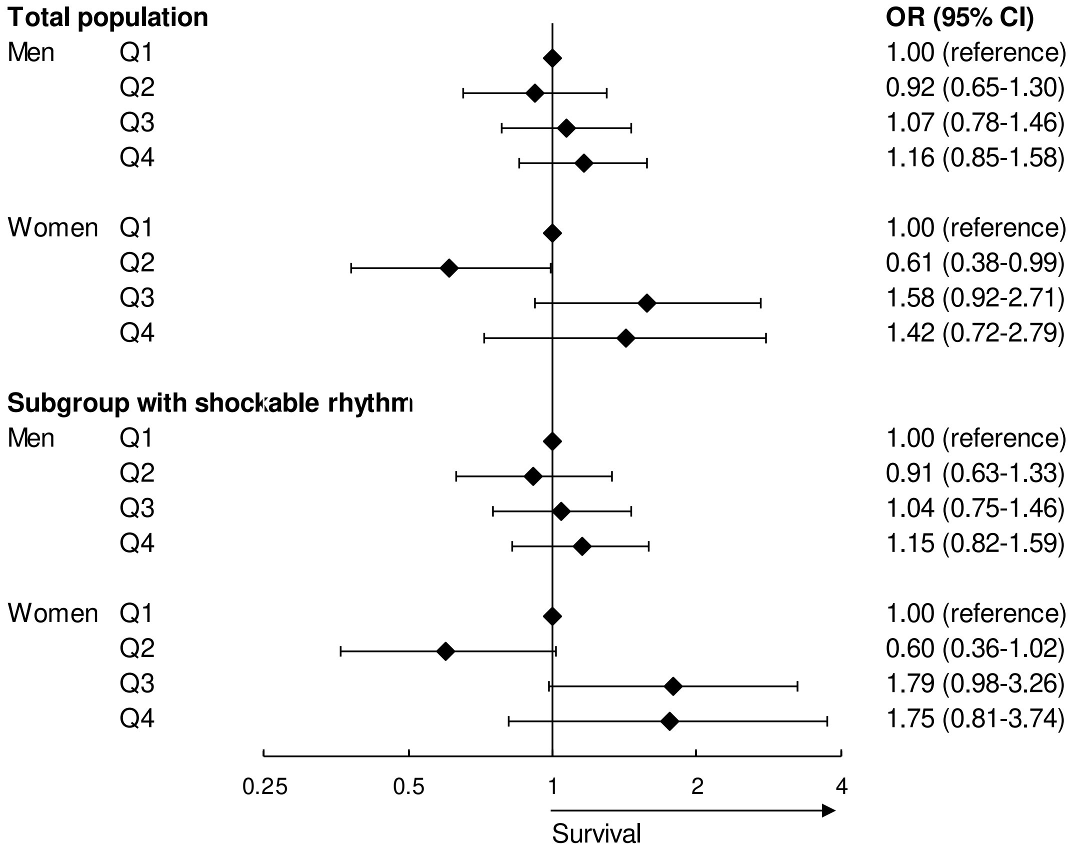

In men, no significant association was observed between increasing quartiles of personal income and OHCA survival. In women, Q2 of personal income was significantly associated with worse survival in comparison to Q1 in the fully adjusted model, but Q3 and Q4 of personal income were not associated with survival.

Subgroup analysis in men and women with a shockable initial rhythm showed small decreases in odds ratios in men and women.

eFigure 3. Distribution of personal income over age in men and women.

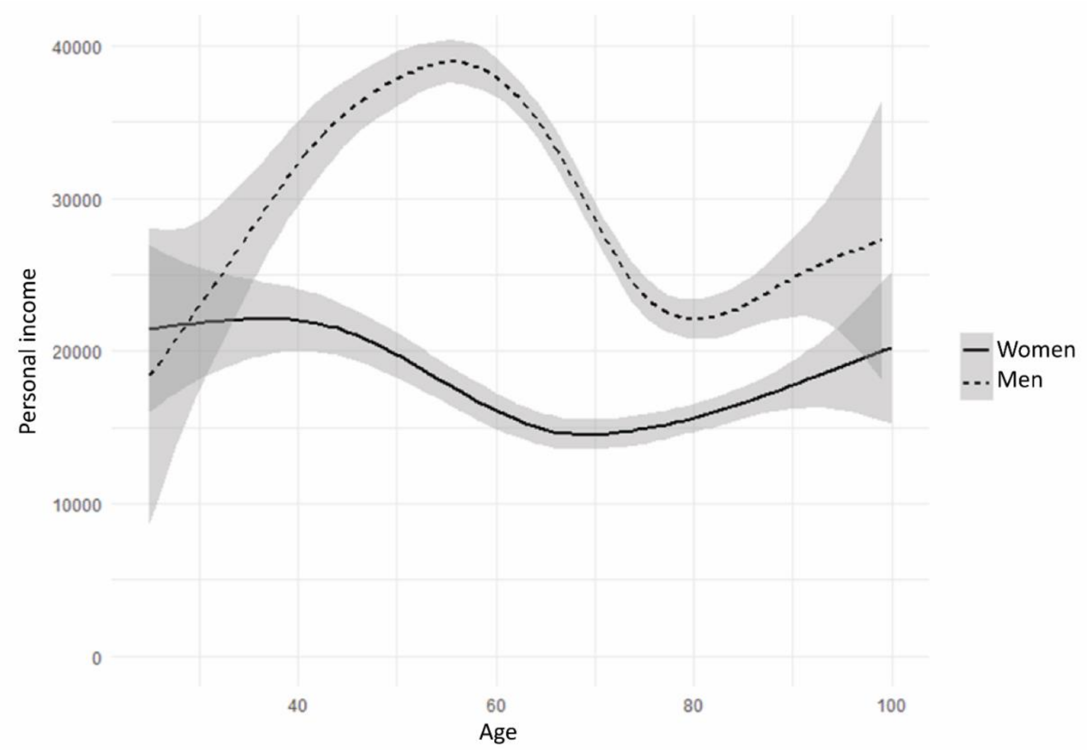

eFigure 4. Distribution of household income over age in men and women.

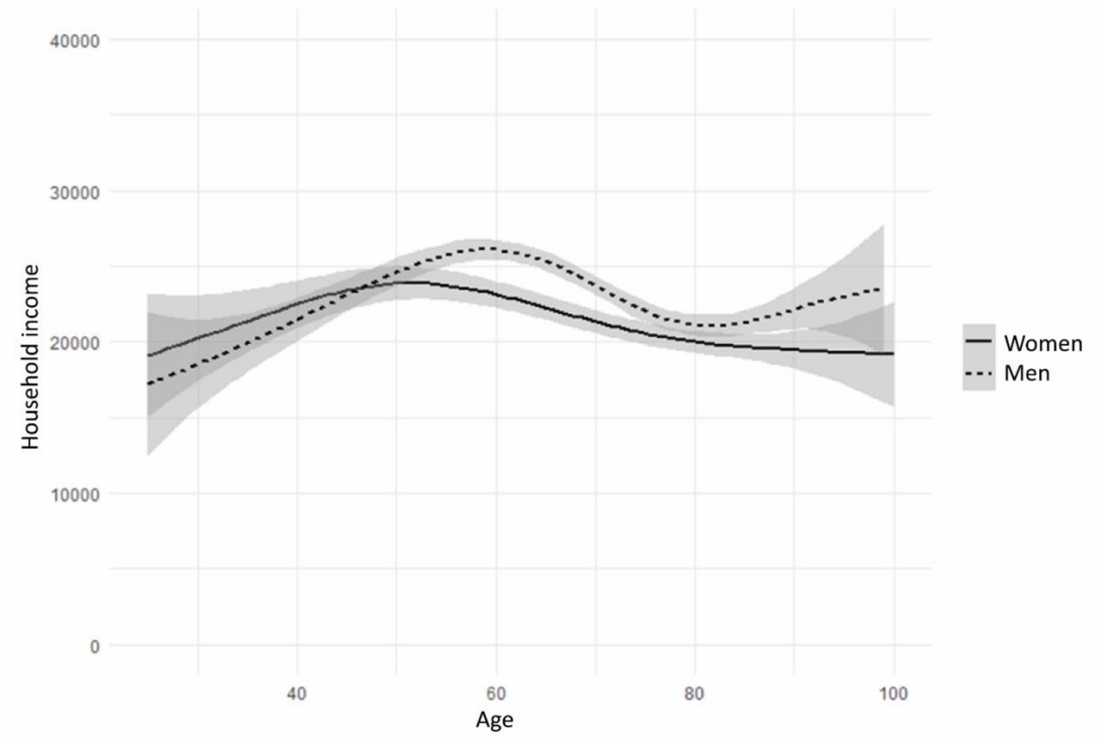

eFigure 5. Forest plot of the association between personal income and OHCA survival in the fully adjusted model in men and women stratified by age.

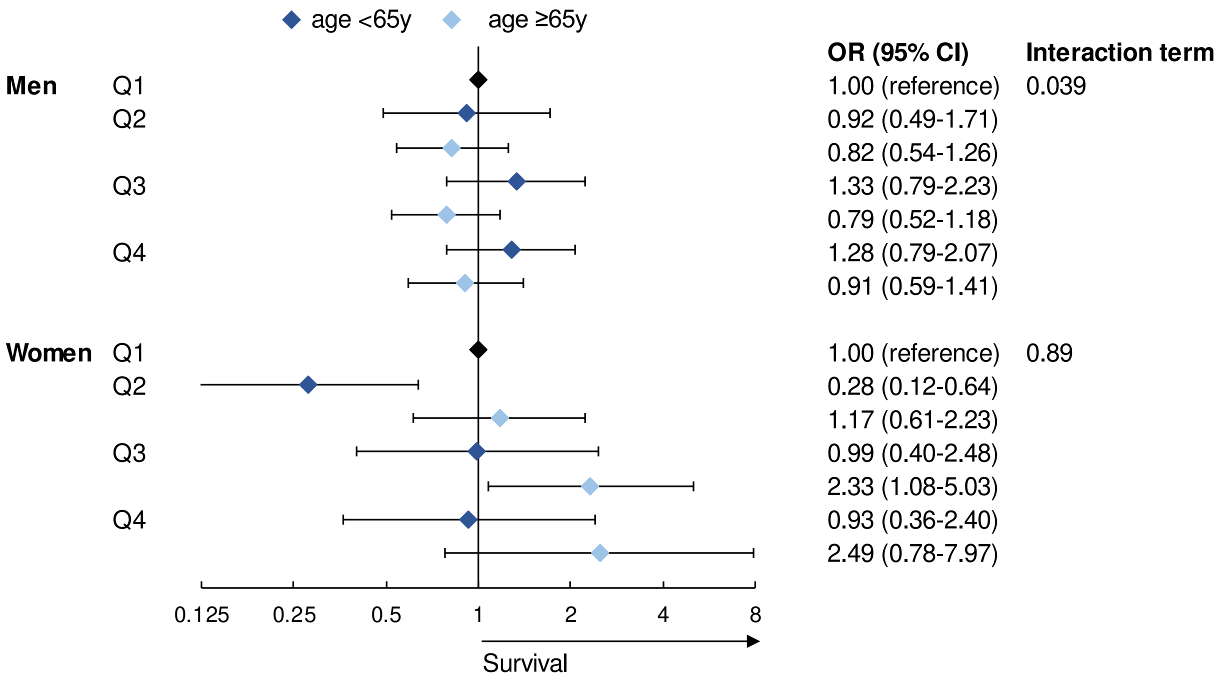

Supplement: Supplementary data [file openhrt-2022-002044supp001.pdf]
